# Magnetic Resonance Enterography Predicts Long‐Term Outcomes in Crohn's Disease: A Systematic Review and Meta‐Analysis

**DOI:** 10.1002/jgh3.70408

**Published:** 2026-04-16

**Authors:** Shanshan Wu, Jiayi Yang, Seniu Jizhi, Aniu Liu, Xingyu Chen, Zhonglu Chen, Shumei Zheng

**Affiliations:** ^1^ Department of Gastroenterology and Hepatology The General Hospital of Western Theater Command Chengdu China; ^2^ Department of Radiology The Affiliated Wuxi People's Hospital of Nanjing Medical University Jiangsu China

**Keywords:** adult patients, clinical outcomes, Crohn's disease, inflammatory bowel disease, magnetic resonance Enterography, meta‐analysis, systematic review

## Abstract

**Background and Aims:**

Crohn's disease (CD) is characterized by chronic transmural inflammation, which often leads to bowel damage. While mucosal healing (MH) is an established treatment target, transmural healing (TH) has emerged as a more comprehensive goal. Magnetic resonance enterography (MRE) provides excellent soft‐tissue resolution for assessing TH. This meta‐analysis evaluates the prognostic value of MRE in predicting surgery, hospitalization, and treatment escalation in adults with CD.

**Methods:**

A total of 16 studies involving adult CD patients with ≥ 12 months of follow‐up (with a mean duration of 4.42 ± 3.90 years) were included if they incorporated pre‐ and posttreatment MRE, reported baseline MRE phenotypes (e.g., penetrating/stenosing lesions), and documented posttreatment TH. Hazard ratios (HRs) were used to compare associations between baseline MRE features, TH achievement, and long‐term outcomes.

**Results:**

Baseline MRE‐defined penetrating lesions were associated with increased risks of surgery (HR = 1.62, 95% CI: 1.37–1.91, *p* < 0.00001) and hospitalization (HR = 1.34, 95% CI: 1.18–1.53, *p* < 0.0001). Similarly, stricturing lesions were associated with an elevated risk of surgery (HR = 1.65, 95% CI: 1.39–1.96, *p* < 0.00001). Achieving TH was linked to lower risks of surgery (HR = 0.55, 95% CI: 0.44–0.68, *p* < 0.00001), hospitalization (HR = 0.54, 95% CI: 0.45–0.64, *p* < 0.00001), and treatment escalation (HR = 0.51, 95% CI: 0.46–0.57, *p* < 0.00001). Notably, TH achieved significantly greater risk reduction than MH alone, as evidenced by two direct comparative studies of these two treatment targets (HR = 0.28 and 0.26, respectively).

**Conclusion:**

MRE‐identified penetrating/stenosing lesions at baseline predict higher risks of surgery and hospitalization. Conversely, TH achievement was associated with a marked reduction in adverse outcomes. Incorporating MRE into CD management facilitates early identification of high‐risk patients for timely treatment intensification, thereby supporting personalized therapeutic strategies.

## 1 Introduction

Inflammatory bowel disease (IBD) primarily includes CD and ulcerative colitis (UC). Its exact etiology and pathogenesis remain incompletely understood. The prevailing view is that IBD results from a persistent, excessive, abnormal inflammatory response in the gut triggered by interactions among environmental, genetic, infectious, and immune factors [1]. In developed western countries, the incidence of IBD has stabilized, with the prevalence exceeding 0.3% in some regions. In newly industrialized countries, the incidence of IBD is increasing but has not yet peaked [2]. These trends indicate that IBD is evolving into a global health concern. CD, characterized by transmural inflammation and discontinuous (skip) lesions, presents greater clinical complexity. It can affect the entire digestive tract from the mouth to the anus, follows a chronic progressive course, and often leads to structural damage, such as intestinal strictures and penetrating lesions (fistulas/abscesses) [3]. These complications lead to recurrent symptoms, an increased need for hospitalization, an increased risk of treatment escalation, and even disabling damage, severely impairing long‐term quality of life [4, 5].

The clinical symptoms of CD do not always correlate with the actual degree of intestinal inflammation [6]. Therefore, targeting clinical remission alone is insufficient to effectively prevent disease progression and complications. With increasing understanding, treatment goals have evolved from clinical remission to MH. Achieving MH in CD patients is associated with significantly reduced risks of surgery and hospitalization [7]. Currently, endoscopy is the primary method for assessing MH but has inherent limitations [8]. First, conventional endoscopy cannot evaluate the entire small intestine, especially when intestinal stenosis occurs, as the endoscope cannot pass through the stenotic segment. Second, endoscopy mainly observes mucosal surface lesions and cannot be used to evaluate transmural inflammation or extraintestinal complications. Finally, endoscopy is an invasive examination that may cause complications such as intestinal bleeding and perforation, limiting its repeated use for disease monitoring. As a noninvasive examination method, cross‐sectional imaging technology can be used to evaluate the entire intestinal tract and extraintestinal lesions. It is a supplement and alternative to endoscopy for disease evaluation and monitoring in patients with CD [9].

CD is inherently a transmural disease. Therefore, achieving MH does not always indicate complete resolution of deep inflammation [10, 11]; persistent deep wall inflammation may still drive disease progression and increase the risk of adverse outcomes [12]. Consequently, the International Organization for the Study of Inflammatory Bowel Disease (IOIBD) has proposed TH as a potential deeper treatment target in long‐term CD management strategies. TH assessment relies primarily on cross‐sectional imaging techniques, such as computed tomography enterography (CTE), intestinal ultrasound (IUS), and MRE. CTE offers rapid imaging and is suitable for acute abdominal presentations, but its main limitation is cumulative radiation exposure, which is particularly concerning in specific populations, such as pregnant women and children [13]. IUS is advantageous because of its convenience, lack of ionizing radiation, low cost, and high patient acceptance [14]. However, image quality can be affected by bowel gas and abdominal adiposity; its ability to assess deeply located lesions and complex extramural complications is limited, and its reliability is highly operator dependent [15]. MRE provides a comprehensive assessment of bowel wall inflammation depth, activity, and complications in patients with CD. Owing to its noninvasive nature, lack of radiation, excellent soft tissue resolution, and multiparametric capabilities, MRE has become a vital tool for CD diagnosis, disease activity assessment, and treatment monitoring [13].

Although the concept of TH based on cross‐sectional imaging as a treatment endpoint is preliminarily accepted, existing studies on TH and long‐term CD outcomes have several limitations, such as small sample sizes, varying follow‐up durations, heterogeneous definitions of TH, and a lack of systematic integration of evidence. This meta‐analysis is the first to systematically evaluate the predictive value of MRE for surgical intervention, hospitalization, and treatment escalation in adult CD patients. These findings provide an evidence base for personalized CD treatment decisions.

## 2 Methods

This meta‐analysis was registered on PROSPERO (CRD42024585848) and adhered to the Preferred Reporting Items for Systematic Reviews and Meta‐Analyses (PRISMA) statement. A review of previously published studies required neither ethical approval nor informed consent.

### 2.1 Literature Search

A comprehensive systematic literature search was performed in PubMed/MEDLINE, Web of Science, Embase, and the Cochrane Library (up to June 1, 2025). Medical subject headings (MeSH) and free‐text terms related to “Magnetic Resonance Enterography,” “Inflammatory Bowel Disease,” and “Crohn's Disease” were used. The search strategies were iteratively adjusted and optimized according to the specific requirements of each database. Additionally, reference lists of eligible studies and review articles were manually checked to identify other potentially relevant publications.

### 2.2 Inclusion Criteria

Studies meeting the following criteria were included: (1) the study population included adult CD patients (age ≥ 18 years) who underwent MRE both before and after treatment; (2) the studies included survival analysis and reported HRs for outcomes such as hospitalization, surgery, and treatment escalation; (3) the mean follow‐up time was ≥ 12 months; and (4) the studies were published in full text in peer‐reviewed journals.

Studies were excluded if they met the following criteria: (1) they were animal experiments, conference abstracts, case reports, commentaries, reviews, letters, or obviously irrelevant studies; (2) they focused on pediatric CD patients; (3) they researched duplicated data; and (4) their full text versions were unavailable or necessary data could not be extracted.

### 2.3 Study Selection

Two researchers (Shanshan Wu and Jiayi Yang) independently screened the literature. After the search, EndNote 2020 software was used for systematic organization and deduplication of retrieved citations to ensure data integrity and consistency. The initial screening was based on titles and abstracts. The full texts of the initially selected articles were subsequently independently reviewed to finalize the studies that met the inclusion criteria.

### 2.4 Definition of Adverse Outcomes in CD Patients

The long‐term adverse outcomes considered in this review were surgery, hospitalization, and treatment escalation during the follow‐up period after treatment, defined as follows: (1) surgery: any CD‐related intestinal surgery during follow‐up, such as abscess drainage, endoscopic dilation, or ileal resection; (2) hospitalization: hospital admission due to disease exacerbation or CD‐related complications during follow‐up; and (3) treatment escalation: measures required due to inadequate disease control with the current regimen, including increasing the dose of existing medication, initiating biologic therapy for the first time, or switching from one biologic agent to another.

### 2.5 Data Extraction and Quality Assessment

During the data extraction phase, researchers independently extracted the core indicators of the study design (including study characteristics, study population, protocol design, follow‐up period, HR value, etc.), and after independent review, a standardized data table was constructed for subsequent statistical analysis. Any disagreements were resolved through discussion until a consensus was reached.

The quality of the included studies was assessed via the Newcastle–Ottawa Scale (NOS). On the basis of the NOS score (range 0–9), studies with scores ≥ 6 were considered high quality, those with scores of 4–5 were considered moderate quality, and those with scores of 0–3 were considered low quality.

### 2.6 Statistical Analysis

The data from each study were transcribed into tables. Frequencies are presented as numbers and percentages, and continuous variables are presented as the means ± standard deviations (M ± SD). To pool continuous variables (e.g., mean age and mean follow‐up time), medians and interquartile ranges/ranges extracted from relevant studies were converted to M ± SD values via formulas provided in Chapter 6.5.2.5 of the Cochrane Handbook. The converted data were then pooled as recommended by the Cochrane Handbook [16].

No CD patients in the included studies experienced primary outcome events at baseline. The primary endpoints were surgery, hospitalization, and treatment escalation (as defined above). The impact of MRE findings on long‐term adverse outcomes was assessed via HR. If reported, the HR and 95% confidence interval (CI) were extracted directly from each study. If both univariate and multivariate analysis results were reported, HRs from multivariate models were preferentially extracted. Finally, HRs were log‐transformed, and the standard error (SE) of the Log HR was calculated for further statistical analysis.

Heterogeneity among studies was quantified via the *I* ^2^ statistic and graded according to the Cochrane Handbook recommendations: *I* ^2^ ≤ 40%, low heterogeneity; 41%–60%, moderate heterogeneity; and 61%–100%, substantial heterogeneity. For subgroups with moderate or substantial heterogeneity, sensitivity analysis was performed by sequentially excluding individual studies and repeating the meta‐analysis to assess the robustness of the results. All the statistical calculations were performed via Review Manager (Rev Man, version 5.3) software. A *p* < 0.05 was considered to indicate statistical significance.

## 3 Results

### 3.1 Characteristics and Quality Assessment of the Included Studies

**FIGURE 1:**
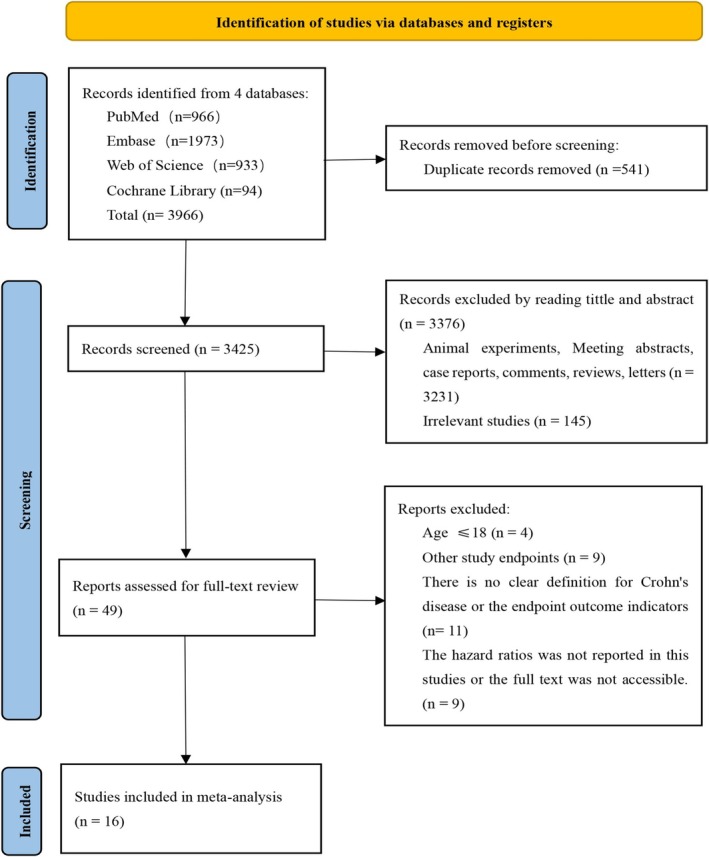
Flowchart of the literature screening process.

Systematic searches of PubMed (*n* = 966), Embase (*n* = 1973), Web of Science (*n* = 933), and the Cochrane Library (*n* = 94) identified 3966 records. Ultimately, 16 cohort studies [10, 17, 18, 19, 20, 21, 22, 23, 24, 25, 26, 27, 28, 29, 30, 31] (Figure 1) were included, consisting of three prospective studies [17, 18, 19] and 3 multicenter studies [17, 20, 21]. These 16 studies included 3249 CD patients with a mean age of 32.91 ± 15.29 years and a mean follow‐up time of 4.42 ± 3.90 years; 56.54% were male. This study focused on prognostic indicators, including surgery, hospitalization, and treatment escalation rates. Eight studies reported only surgery or treatment escalation as the primary outcome, whereas eight studies assessed multiple prognostic indicators for a more comprehensive evaluation. The basic characteristics of the included studies are shown in Table 1. The NOS score for all the studies was ≥ 7, indicating good quality, as shown in Table 2. The extracted HR values are shown in Table 3.

**TABLE 1:** Study characteristics of the included cohort studies.

First author (publication year)	Study design	Single‐center or multicenter study	NOS score	Country	Sample size (male)	Age (year)	Study duration (year)
Sagami S et al. 2019 [10]	R	S	7	Japan	50 (34)	34.41 ± 3.57	1.26 ± 0.41
Fiorino G et al. 2017 [17]	P	M	8	Italy	142 (67)	30.63 ± 13.17	6.28 ± 5.77
Takenaka K et al. 2023 [18]	P	S	8	Japan	134 (99)	34.11 ± 13.49	8.05 ± 6.74
Takenaka K et al. 2017 [19]	P	S	9	Japan	200 (137)	33.83 ± 10.84	1.85 ± 0.81
Fernandes SR et al. 2023 [20]	R	M	7	Portugal	404 (191)	34.35 ± 15.62	5.0
Revés J et al. 2025 [21]	R	M	9	Portugal	154 (75)	26.17 ± 3.02	1.00 ± 0.50
Deepak P et al. 2018 [22]	R	S	9	USA	150 (75)	25.11 ± 10.48	4.39 ± 4.04
Lee JH et al. 2018 [23]	R	S	8	South Korea	173 (127)	22.05 ± 6.73	1.37 ± 0.66
Lu Y et al. 2024 [24]	R	S	9	China	175 (115)	30.00 ± 2.60	1.46 ± 0.22
Stidham RW et al. 2016 [25]	R	S	9	USA	221 (92)	40.96 ± 15.68	3.30 ± 1.43
Perl D et al. 2019 [26]	R	S	9	USA	157 (65)	31.03 ± 14.88	2.0
Grass F et al. 2019 [27]	R	S	8	USA	559 (295)	40.40 ± 20.29	9.07 ± 2.62
Deepak P et al. 2016 [28]	R	S	8	USA	150 (75)	25.11 ± 10.48	9.26 ± 3.41
Lafeuille P et al. 2021 [29]	R	S	7	France	154 (113)	28.2 ± 14.4	2.45 ± 0.62
Oh K et al. 2022 [30]	R	S	8	South Korea	392 (261)	NA	1.50 ± 0.37
Gallego JC et al. 2019 [31]	R	S	8	Spain	34 (16)	36.78 ± 11.24	5.17 ± 1.55

Abbreviations: M, multicenter study; P, prospective cohort; R, retrospective cohort; S, Single‐center.

**TABLE 2:** Newcastle–Ottawa Scale scores.

Study	Selection				Comparability		Exposure/outcome			Total
First author (publication year)	The cohort was truly or somewhat representative of a typical adult Crohn's disease cohort	Selection of the control subjects was from the same community as the adult Crohn's disease cohort	Ascertainment of adult Crohn's disease was made via secure record OR structured interview	Demonstration that outcome of interest was not present at start of study	Cohorts are comparable based on design or control for major confounders	Cohorts are comparable based on design or control for minor confounders	Assessment of outcome was independent OR linked to medical records	Follow‐up time was clearly defined	Follow‐up was adequate (all subjects accounted for or ≤ 20% attrition)	Total
Sagami S et al. 2019 [10]	1	1	1	1	0	0	1	1	1	7
Fiorino G et al. 2017 [17]	1	1	1	1	1	1	0	1	1	8
Takenaka K et al. 2023 [18]	0	1	1	1	1	1	1	1	1	8
Takenaka K et al. 2017 [19]	1	1	1	1	1	1	1	1	1	9
Fernandes SR et al. 2023 [20]	1	1	1	1	0	1	0	1	1	7
Revés J et al. 2025 [21]	1	1	1	1	1	1	1	1	1	9
Deepak P et al. 2018 [22]	1	1	1	1	1	1	1	1	1	9
Lee JH et al. 2018 [23]	1	1	1	0	1	1	1	1	1	8
Lu Y et al. 2024 [24]	1	1	1	1	1	1	1	1	1	9
Stidham RW et al. 2016 [25]	1	1	1	1	1	1	1	1	1	9
Perl D et al. 2019 [26]	1	1	1	1	1	1	1	1	1	9
Grass F et al. 2019 [27]	1	1	1	1	1	1	0	1	1	8
Deepak P et al. 2016 [28]	1	1	1	0	1	1	1	1	1	8
Lafeuille P et al. 2021 [29]	1	1	1	1	1	1	0	1	1	8
Oh K et al. 2022 [30]	1	1	1	0	1	1	1	1	1	8
Gallego JC et al. 2019 [31]	1	1	1	1	1	1	0	1	1	8

**TABLE 3:** Summary of correlations between MRE‐related parameters and adverse outcomes in adults with Crohn's disease.

Study	MRE parameter	Measure of effect	Effect size	*p*
*Any CD‐related surgery*				
Fiorino G et al. 2017 [17]	Intestinal stenosis, fistula or abscess on baseline scanning	Multivariable HR	3.21 (1.87–5.53)	*p* ≤ 0.001
Deepak P et al. 2018 [22]	Intestinal stenosis, fistula or abscess on baseline scanning	Multivariable HR	2.20 (1.20–4.10)	*p* = 0.010
Lee JH et al. 2018 [23]	Intestinal stenosis, fistula or abscess on baseline scanning	Multivariable HR	5.45 (1.10–27.18)	*p* = 0.015
Lu Y et al. 2024 [24]	Intestinal stenosis, fistula or abscess on baseline scanning	Multivariable HR	9.17 (1.82–46.1)	*p* = 0.007
Takenaka K et al. 2017 [19]	Small bowel dilation > 20 mm	Multivariable HR	10.99 (3.13–38.56)	*p* < 0.001
Stidham RW et al. 2016 [25]	Small bowel dilation > 35 mm	Multivariable HR	2.92 (1.73–4.94)	*p* ≤ 0.001
Perl D et al. 2019 [26]	Small bowel dilation > 30 mm	Multivariable HR	2.19 (1.02–4.68)	*p* = 0.044
Grass F et al. 2019 [27]	Small bowel dilation > 30 mm	Multivariable HR	3.06 (2.06–4.55)	*p* < 0.0001
Takenaka K et al. 2023 [18]	TH^c^	Multivariable HR	0.02 (0.001–0.92)	*p* = 0.040
Fernandes SR et al. 2023 [20]	TH^b^	Multivariable HR	0.16 (0.04–0.67)	*p* = 0.012
Revés J et al. 2025 [21]	TH^a^	Multivariable HR	0.21 (0.05–0.88)	*p* = 0.030
Deepak P et al. 2016 [28]	TH^a^	Multivariable HR	0.34 (0.18–0.63)	*p* < 0.001
Lafeuille P et al. 2021 [29]	TH^a^	Multivariable HR	0.08 (0.01–0.58)	*p* = 0.013
*Hospitalization*				
Fiorino G et al. 2017 [17]	Intestinal stenosis, fistula or abscess on baseline scanning	Multivariable HR	1.88 (1.25–2.83)	*p* = 0.002
Lee JH et al. 2018 [23]	Intestinal stenosis, fistula or abscess on baseline scanning	Multivariable HR	2.75 (1.20–6.34)	*p* = 0.017
Deepak P et al. 2016 [28]	Intestinal stenosis, fistula or abscess on baseline scanning	Multivariable HR	1.87 (1.09–3.23)	*p* = 0.020
Takenaka K et al. 2023 [18]	TH^c^	Multivariable HR	0.11 (0.04–0.32)	*p* < 0.010
Fernandes SR et al. 2023 [20]	TH^b^	Multivariable HR	0.30 (0.15–0.62)	*p* = 0.001
Lu Y et al. 2024 [24]	TH^a^	Multivariable HR	0.21 (0.06–0.69)	*p* = 0.010
Deepak P et al. 2016 [28]	TH^a^	Multivariable HR	0.28 (0.15–0.50)	*p* < 0.001
*Treatment escalation*				
Sagami S et al. 2019 [10]	TH^d^	Multivariable HR	0.24 (0.06–0.92)	*p* = 0.012
Fernandes SR et al. 2023 [20]	TH^b^	Multivariable HR	0.20 (0.13–0.33)	*p* < 0.001
Revés J et al. 2025 [21]	TH^a^	Multivariable HR	0.35 (0.14–0.88)	*p* = 0.020
Lee JH et al. 2018 [23]	TH^a^	Multivariable HR	0.16 (0.04–0.70)	*p* = 0.029
Lu Y et al. 2024 [24]	TH^a^	Multivariable HR	0.14 (0.05–0.38)	*p* < 0.001
Deepak P et al. 2016 [28]	TH^a^	Multivariable HR	0.37 (0.21–0.64)	*p* < 0.001
Lafeuille P et al. 2021 [29]	TH^a^	Multivariable HR	0.15 (0.07–0.32)	*p* < 0.001
Oh K et al. 2022 [30]	TH^a^	Multivariable HR	0.11 (0.06–0.23)	*p* < 0.001
Gallego JC et al. 2019 [31]	TH^c^	Multivariable HR	0.26 (0.07–0.95)	*p* = 0.027

^a^ TH was defined as bowel wall thickness (BWT) ⩽ 3 mm with no inflammatory signs (i.e., ulcer, intramural edema, diffusion‐weighted hyperintensity, and contrast enhancement) and no complications (i.e., abscess, strictures, or fistula).

^b^ TH was defined as bowel wall thickness (BWT) ⩽ 3 mm and no diffusion‐weighted hyperintensity.

^c^ TH was defined as the MRE score of the most affected segment < 2.

^d^ TH was defined as a MaRIA score of < 50.

### 3.2 Risk of Surgery

#### 3.2.1 Association Between Baseline Penetrating Lesions and Surgical Risk

**FIGURE 2:**
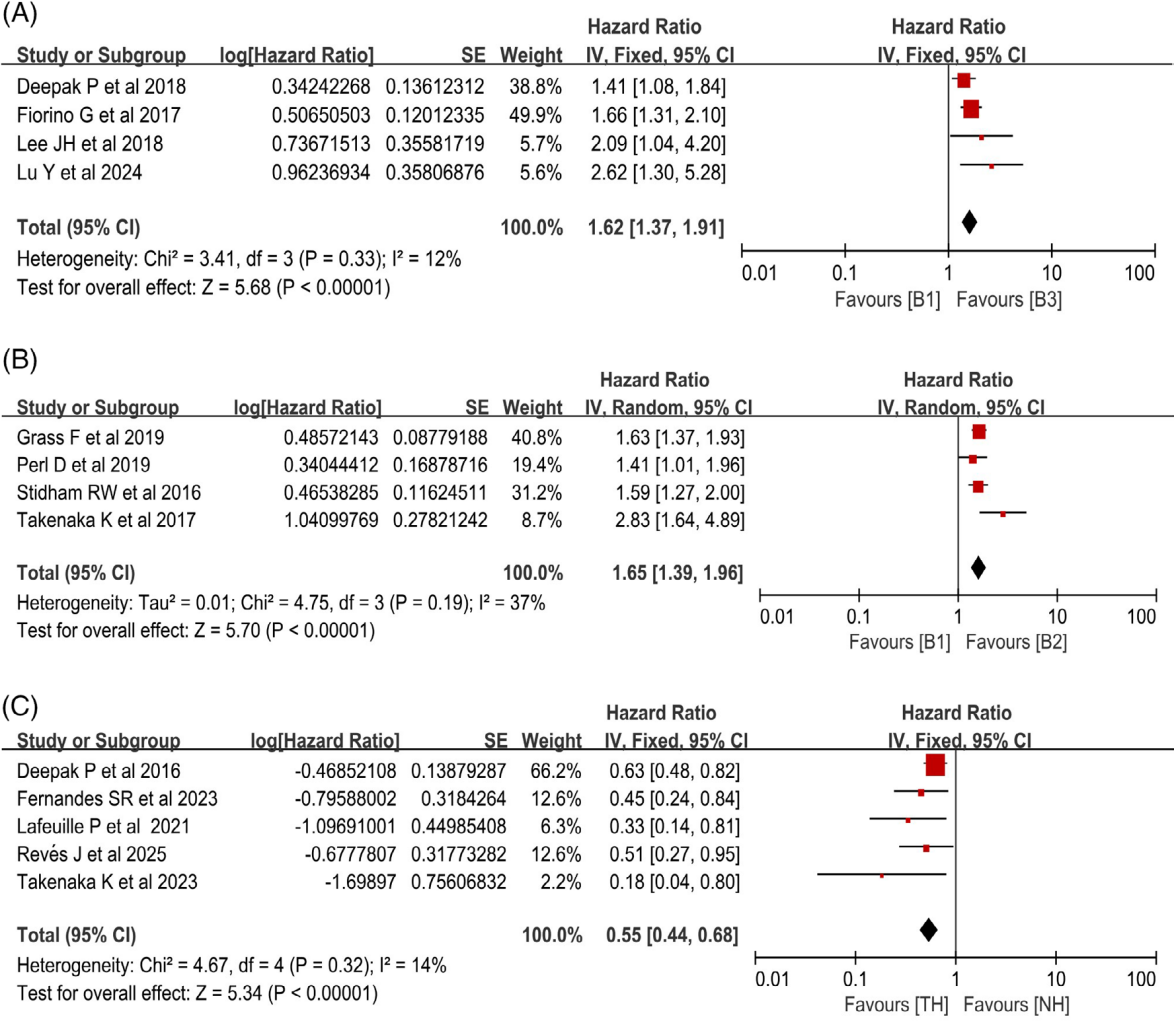
Forest plots for the association of baseline MRE phenotypes and posttreatment TH with surgery in CD. (A) Penetrating lesions. (B) Stricturing lesions. (C) TH.

Among the 16 studies included, 4 analyzed the impact of baseline MRE‐defined penetrating lesions (B3) on surgical risk during the follow‐up period in patients with CD [17, 22, 23, 24]. Heterogeneity testing indicated low heterogeneity among the studies (*χ* ^2^ = 3.41, df = 3, *p* = 0.33; *I* ^2^ = 12%), so a fixed‐effects model was used to pool the HRs. Statistical analysis revealed that the presence of baseline MRE‐defined penetrating lesions was associated with an increased risk of surgery during follow‐up in CD patients (HR = 1.62, 95% CI: 1.37–1.91, *p* < 0.00001) (Figure 2A).

#### 3.2.2 Association Between Baseline Stricture Lesions and Surgical Risk

Among the 16 included studies, 4 studies analyzed the impact of baseline MRE‐defined stricturing lesions (B2) and the risk of surgery during follow‐up [19, 25, 26, 27]. Heterogeneity testing indicated low heterogeneity (*χ* ^2^ = 4.75, df = 3, *p* = 0.19, *I* ^2^ = 37%), which was considered acceptable. Given that this subgroup had relatively greater heterogeneity (*I* ^2^ = 37%) than other subgroups did, a more conservative random effects model was used for pooling. Baseline MRE‐defined stricturing lesions in CD patients were observed to be linked with higher rates of surgery over the follow‐up period. (HR = 1.65, 95% CI: 1.39–1.96, *p* < 0.00001) (Figure 2B). Sensitivity analysis suggested that heterogeneity might stem from variations in the morphological criteria for small bowel stenosis with prestenotic dilation among the studies; the four studies used different thresholds for luminal dilation: > 20 mm (*n* = 1), > 30 mm (*n* = 2), and > 35 mm (*n* = 1) (Table 3). This inconsistency in diagnostic thresholds may introduce potential bias in the effect size.

#### 3.2.3 Association Between Achievement of TH Posttreatment and Surgical Risk

Among the 16 studies included, 5 analyzed the impact of achieving TH after treatment on surgical risk during the follow‐up period in patients with CD [18, 20, 21, 28, 29]. Heterogeneity testing indicated low heterogeneity among the studies (*χ* ^2^ = 4.67, df = 4, *p* = 0.32; *I* ^2^ = 14%), so a fixed‐effects model was used. The results revealed that CD patients who achieved TH after treatment were associated with a lower risk of intestinal surgery during follow‐up (HR = 0.55, 95% CI: 0.44–0.68, *p* < 0.00001) (Figure 2C).

### 3.3 Risk of Hospitalization

#### 3.3.1 Association Between Baseline Penetrating Lesions and the Risk of Hospitalization

**FIGURE 3:**
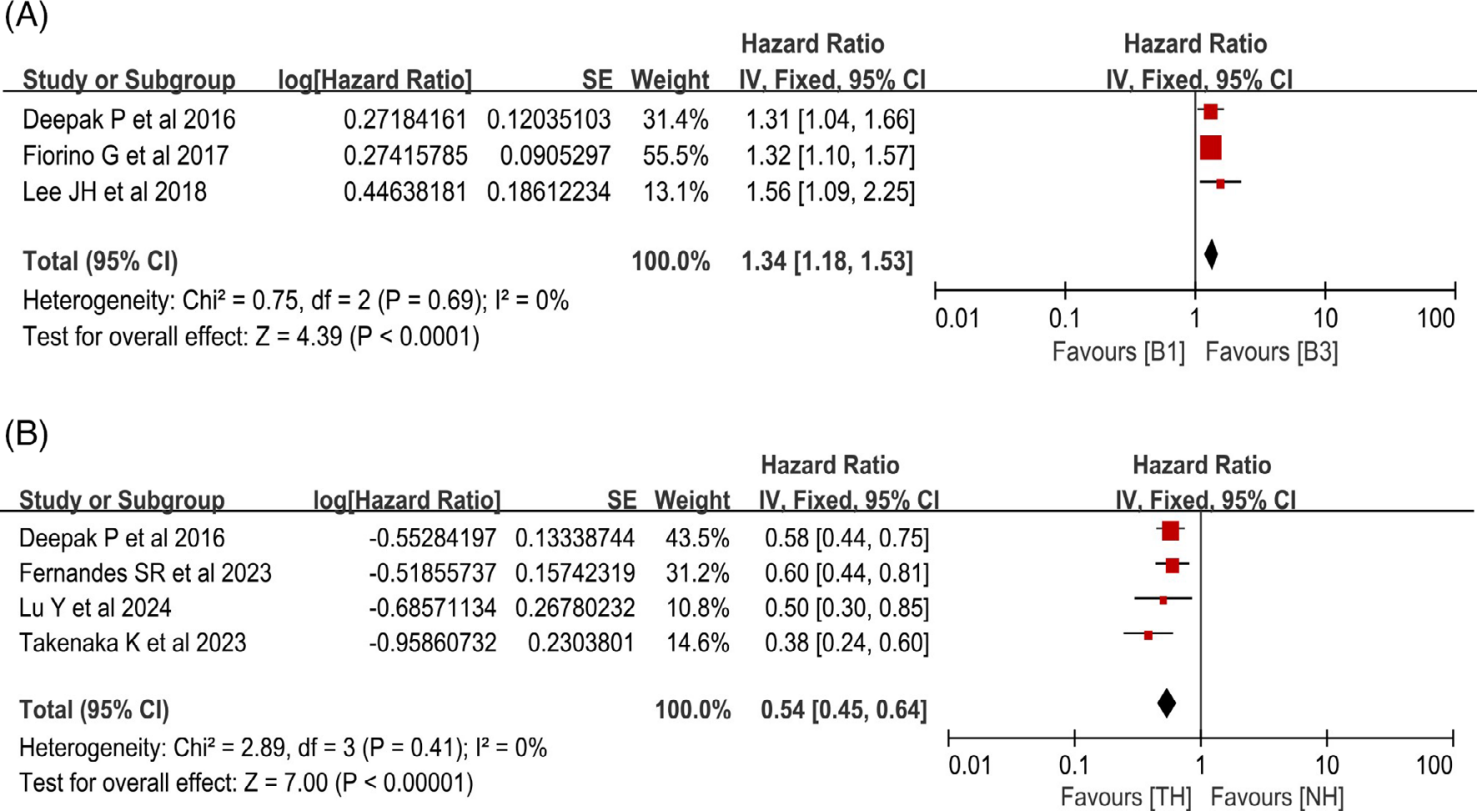
Forest plots for the association of baseline MRE phenotypes and posttreatment TH with hospitalization in CD. (A) Penetrating lesions. (B) TH.

Among the 16 studies included, 3 analyzed the impact of baseline MRE‐defined penetrating lesions (B3) on the risk of hospitalization in patients with CD during the follow‐up period [17, 23, 28]. Heterogeneity testing indicated low heterogeneity among the studies (*χ* ^2^ = 0.75, df = 2, *p* = 0.69; *I* ^2^ = 0%), so a fixed‐effects model was used to pool the HRs. The results showed that baseline MRE‐defined penetrating lesions were associated with a higher risk of hospitalization during the follow‐up period in CD patients. (HR = 1.34, 95% CI: 1.18–1.53, *p* < 0.0001) (Figure 3A).

#### 3.3.2 Association Between Achievement of TH Posttreatment and Hospitalization Risk

Among the 16 studies included, 4 analyzed the impact of achieving TH after treatment on hospitalization risk during the follow‐up period in patients with CD [18, 20, 24, 28]. Heterogeneity testing indicated low heterogeneity among the studies (*χ* ^2^ = 2.89, df = 3, *p* = 0.41; *I* ^2^ = 0%), so a fixed‐effects model was used. Statistical analysis revealed that compared with NH patients, CD patients who achieved TH after treatment were associated with lower rates of hospitalization during follow‐up (HR = 0.54, 95% CI: 0.45–0.64, *p* < 0.00001) (Figure 3B).

### 3.4 Risk of Treatment Escalation

#### 3.4.1 Association Between Achievement of TH Posttreatment and Treatment Escalation Risk

**FIGURE 4:**
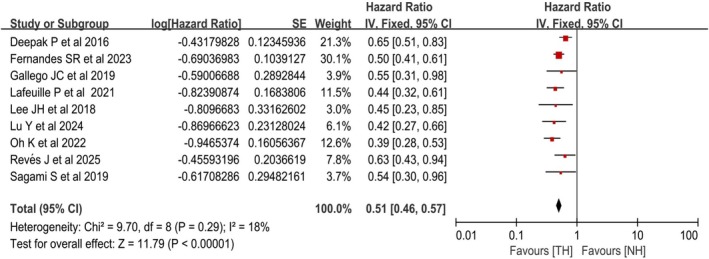
Forest plot for the association of posttreatment TH with treatment escalation in CD.

Among the 16 studies included, 9 cohort studies analyzed the impact of achieving TH after treatment on the risk of treatment escalation in CD patients during the follow‐up period [10, 20, 21, 23, 24, 28, 29, 30, 31]. Heterogeneity testing indicated low heterogeneity among the studies (*χ* ^2^ = 9.70, df = 8, *p* = 0.29; *I* ^2^ = 18%), so a fixed‐effects model was used. The results revealed that CD patients achieving TH after treatment were observed to have lower rates of treatment escalation during follow‐up (HR = 0.51, 95% CI: 0.46–0.57, *p* < 0.00001) (Figure 4).

## 4 Discussion

This meta‐analysis is the first to systematically quantify the predictive value of MRE for long‐term outcomes in adult CD patients based on a large sample size. We found that baseline MRE‐defined penetrating lesions were significantly associated with a higher risk of surgical intervention during follow‐up in this patient population. Penetrating lesions are among the most severe complications of CD, often leading to fistulas and abscesses, which cause persistent inflammation, infection, or malnutrition; severely impact quality of life; and often necessitate urgent treatment escalation and surgical intervention [32]. Although biologics can induce fistula closure in some patients, approximately half ultimately require surgery. Delayed intervention may increase the risks of postoperative recurrence and reoperation [33]. Therefore, early detection of penetrating lesions is crucial for guiding clinical decisions. Cross‐sectional imaging, such as MRE, allows comprehensive assessment of full‐thickness inflammation and extramural complications, providing a “therapeutic window” for initiating intensive treatment promptly and considering surgical intervention if medical therapy fails, with the goal of delaying or halting disease progression. Furthermore, this meta‐analysis revealed that baseline MRE‐defined stricturing lesions were associated with a markedly higher risk of subsequent surgery. A previous study of 557 IBD patients revealed that approximately 33% of CD patients had small bowel strictures (two‐thirds were inflammatory) [34]. Without timely monitoring and intervention, inflammatory strictures may progress to irreversible fibrotic strictures. Owing to the lack of safe and effective antifibrotic drugs, these patients often experience repeated hospitalizations and surgeries [35]. These findings suggest that early risk stratification and prognosis prediction on the basis of MRE phenotypes may guide clinicians in initiating early intensive therapy and improving disease outcomes.

Currently, the long‐term treatment target for CD remains MH. Achieving MH significantly improves long‐term prognosis [36]. However, CD is a transmural inflammatory disease. Studies indicate that the disease may progress even after MH is achieved [37]. Therefore, the STRIDE‐II consensus proposed TH as a potential deeper treatment target [38]. This meta‐analysis, through data integration, demonstrated that achieving TH after treatment was associated with lower risks of surgery, hospitalization, and treatment escalation compared with NH. Currently, studies directly comparing MH and TH are limited. Four studies included in this meta‐analysis directly compared the prognostic value of TH with that of MH, consistently demonstrating stronger predictive power for TH. For example, Takenaka et al. [18] reported that compared with those who achieved only MH, CD patients who achieved TH had significantly lower risks of hospitalization (HR = 0.11 vs. 0.39) and surgery (HR = 0.02 vs. 0.19) during follow‐up. Lafeuille et al. [29] also reported that patients who achieved TH had far lower risks of surgery, hospitalization, and treatment escalation than those who achieved only MH did (HR = 0.28, 95% CI: 0.00–0.74, *p* = 0.01). Fernandes et al. [20] reported that the risk of surgery was significantly lower in the TH group than in the NH group (HR = 0.160, 95% CI: 0.038–0.673; *p* = 0.012), whereas no significant difference was found between the MH and NH groups. Additionally, Oh et al. [30] reported that compared with achieving TH, achieving only MH was associated with greater risks of surgery, treatment escalation, and hospitalization (HR = 3.903, 95% CI: 1.635–9.315, *p* = 0.002). In summary, TH represents a treatment goal beyond MH. Achieving TH was associated with lower rates of complications and adverse outcomes.

Although targeting TH holds clinical value, its application in clinical practice faces challenges. On the one hand, reported rates of TH achievement remain relatively low, ranging between 14% and 42.4% [39], reflecting the complexity of CD treatment. Potential reasons include the following: current medications may not fully resolve transmural inflammation in all patients; the varying timing of posttreatment MRE assessment may lead to underestimation of TH rates; and the limited availability, complexity, and increased cost of advanced cross‐sectional imaging, such as MRE, restrict its routine use and the widespread implementation of TH as a target. On the other hand, there is currently no unified standard for MRE assessment of TH. Different scoring systems (Maria, SMaria, Clermont, and C rating systems) vary in imaging features, weightings, and threshold settings, limiting comparability across studies [8, 40].

The definition of TH also lacks uniformity across different cross‐sectional imaging modalities (Table 4). TH assessed by CTE/MRE may focus on normalization of bowel wall thickness and enhancement, whereas IUS‐assessed TH may emphasize normalization of wall thickness, stratification, and blood flow signals [41]. Substantial heterogeneity in TH definitions across studies introduces ambiguity to clinical decision‐making. Simplified TH criteria (e.g., bowel wall thickness [BWT] ≤ 3 mm alone) yield higher TH rates but risk underestimating persistent transmural inflammation, which may lead to premature treatment de‐escalation and elevated relapse risk. In contrast, extended criteria (e.g., BWT ≤ 3 mm plus absence of hypervascularization signs) reduce observed TH rates while improving therapeutic precision and mitigating progression of bowel damage. Although the prognostic value of TH has been well established, its implications need to be interpreted with caution in clinical practice.

**TABLE 4:** Comparison of definitions for transmural healing as assessed by MRE.

Study (Author, Year)	Design of the study	Imaging modality	TH definition
Sagami S et al. 2019 (*n* = 50) [10]	Retrospective cohort; single‐center	MRE	MaRIA score < 50
Takenaka K et al. 2023 (*n* = 134) [18]	Prospective cohort; single‐center	MRE	MaRIA score of the most affected segment < 2
Gallego JC et al. 2019 (*n* = 34) [31]	Retrospective cohort; single‐center	MRE	MaRIA score of the most affected segment < 2
Fernandes SR et al. 2023 (*n* = 404) [20]	Retrospective cohort; multicenter	MRE	BWT ≤ 3 mm Absence of diffusion‐weighted hyperintensity
Revés J et al. 2025 (*n* = 154) [21]	Retrospective cohort; multicenter	MRE	BWT ≤ 3 mm No inflammatory signs (ulcer, edema, enhancement) No complications (abscess, stricture, fistula)
Lee JH et al. 2018 (*n* = 173) [23]	Retrospective cohort; single‐center	MRE	BWT ≤ 3 mm No inflammatory signs (ulcer, edema, enhancement) No complications (abscess, stricture, fistula)
Lu Y et al. 2024 (*n* = 175) [24]	Retrospective cohort; single‐center	MRE	BWT ≤ 3 mm No inflammatory signs (ulcer, edema, enhancement) No complications (abscess, stricture, fistula)
Deepak P et al. 2016 (*n* = 150) [28]	Retrospective cohort; single‐center	MRE	BWT ≤ 3 mm No inflammatory signs (ulcer, edema, enhancement) No complications (abscess, stricture, fistula)
Lafeuille P et al. 2021 (*n* = 154) [29]	Retrospective cohort; single‐center	MRE	BWT ≤ 3 mm No inflammatory signs (ulcer, edema, enhancement) No complications (abscess, stricture, fistula)
Oh K et al. 2022 (*n* = 392) [30]	Retrospective cohort; single‐center	MRE	BWT ≤ 3 mm No inflammatory signs (ulcer, edema, enhancement) No complications (abscess, stricture, fistula)

*Note:* BWT, bowel wall thickness; MaRIA, magnetic resonance index of activity; MRE, magnetic resonance enterography; TH, transmural healing.

Several limitations of this study should be considered when interpreting the results. First, 13 of the 16 included studies were retrospective, potentially introducing selection and information bias that could affect the robustness of the results. Second, the included CD patients were adults; the applicability of these findings to special populations, such as children and pregnant women, requires further validation.

In conclusion, CD is a chronic progressive disorder characterized by cumulative bowel damage and disability. The ultimate therapeutic goal is sustained deep remission. Baseline MRE‐defined penetrating or stricturing lesions were associated with higher risks of hospitalization and surgery. Conversely, achieving TH was associated with lower risks of surgery, hospitalization, and treatment escalation, suggesting that TH may represent a potentially deeper therapeutic target than MH. Cross‐sectional imaging may facilitate early disease phenotyping, risk stratification, and longitudinal dynamic monitoring, thereby supporting timely treatment adjustment to reduce complication risks and improve long‐term prognosis. Future efforts should focus on the standardization of imaging criteria for TH and the optimization of assessment time points to further enhance their clinical applicability.

## Funding

The authors have nothing to report.

## Conflicts of Interest

The authors declare no conflicts of interest.

## Data Availability

The data that support the findings of this study are openly available in Figshare at https://doi.org/10.6084/m9.figshare.31959732.
